# State abortion restrictiveness and prevalence of intimate partner violence and domestic violence among recently birthing black and white individuals

**DOI:** 10.3389/frph.2025.1535865

**Published:** 2025-04-14

**Authors:** Katherine Neff, Stephanie V. Hall, Rieham Owda, Andrea Pangori, Kara Zivin, Angela Montoya, Leila McDonnaugh-Eaddy, Yasamin Kusunoki, April M. Zeoli, Kamilah Davis-Wilson, Anna Courant, Vanessa K. Dalton

**Affiliations:** ^1^ Department of Obstetrics and Gynecology, University of Michigan, Ann Arbor, MI, United States; ^2^ Program on Women’s Healthcare Effectiveness Research, University of Michigan, Ann Arbor, MI, United States; ^3^ Department of Obstetrics and Gynecology, Corewell Health William Beaumont University Hospital, Royal Oak, MI, United States; ^4^ Department of Psychiatry, University of Michigan, Ann Arbor, MI, United States; ^5^ Department of Health Management and Policy, University of Michigan School of Public Health, Ann Arbor, MI, United States; ^6^ Center for Clinical Management Research (CCMR), VA Ann Arbor Healthcare System, Ann Arbor, MI, United States; ^7^ Institute for Healthcare Policy and Innovation, University of Michigan, Ann Arbor, MI, United States; ^8^ College of Human Medicine, Michigan State University, East Lansing, MI, United States; ^9^ Department of Biology, Spelman College, Atlanta, GA, United States; ^10^ Department of Systems, Populations and Leadership, University of Michigan School of Nursing, Ann Arbor, MI, United States; ^11^ Population Studies Center, Institute for Social Research, University of Michigan, Ann Arbor, MI, United States; ^12^ Institute for Firearm Injury Prevention, University of Michigan, Ann Arbor, MI, United States

**Keywords:** intimate partner violence, domestic violence, abortion, policy, health disparities

## Abstract

**Objectives:**

Intimate partner violence (IPV) and non-intimate domestic violence (DV) during pregnancy may result in poor maternal and infant health outcomes. Whether state-level abortion restrictions, enacted by many states even prior to the 2022 *Dobbs v. Jackson Women's Health Organization* decision, are associated with IPV/DV remains unknown. This study aimed to investigate the relationship between IPV/DV during pregnancy and abortion restrictions among Black and White birthing people.

**Study design:**

We analyzed 2020 data from 36 states participating in the CDC Pregnancy Risk Assessment Monitoring System representing 1,931,458 deliveries of which 1,368,237 deliveries (70.84%) are from Black and White birthing individuals. We divided states into restrictive (*N* = 17) and less restrictive (*N* = 19) based on a modified Guttmacher Abortion Policy Hostility Index. We used weighted logistic regression to assess the relationship between state abortion restrictiveness and self-reported IPV/DV.

**Results:**

Overall, birthing individuals in restrictive states had higher odds of reporting IPV/DV during pregnancy than those in less restrictive states (aOR: 1.36, 95% CI: 1.15–1.60). Within racial groups, we found that Black birthing individuals in restrictive states had higher odds of reporting IPV/DV than Black birthing individuals in less restrictive states (aOR:1.75, 95% CI: 1.24–2.47). We saw a similar relationship for White birthing individuals (aOR:1.50, 95% CI: 1.17–1.94).

**Discussion:**

Even when access to abortion was federally protected, individuals in restrictive states had higher odds of experiencing IPV/DV than those in less restrictive states, particularly among Black individuals. These findings suggest possible detrimental impacts of abortion restrictions and their potential to worsen existing health inequities.

## 1 Introduction

Nearly 10% of pregnant people report experiencing either physical or sexual violence during their pregnancy, and Black individuals experience intimate partner violence (IPV) rates (1) at least twice as high as White individuals (2). IPV and non-intimate domestic violence (DV) during pregnancy have serious maternal and infant health consequences, including pregnancy loss, premature birth, low birthweight, and infant mortality (3, 4)**—**outcomes that disproportionately impact Black birthing people and infants (5). Pregnancy is also a period of heightened risk. Pregnant or recently pregnant individuals are more likely to die by homicide than of the three most common obstetric causes of death (hypertensive disorders, hemorrhage, sepsis) (6), and rates are increasing (7). Female victims of homicide are disproportionately more likely to be Black, especially in pregnancy-associated homicides; between 2008 and 2019, nearly 50% of pregnancy-associated homicide victims were Black (8). IPV also precipitates half of pregnancy-associated suicides (2, 6, 9).

Prior research has described the complicated relationships among pregnancy intention, abortion access, and IPV. Compared to individuals with intended pregnancies, people with unwanted or unplanned pregnancies are four times more likely to experience IPV (10) and individuals victimized by IPV are three times more likely to have an abortion during their lifetime (11, 12). Partner conflict or IPV is often a factor in the decision to have an abortion, and those who obtain abortions end their abusive relationships faster and experience less partner violence than those who give birth (13). The reported relationships among pregnancy intention, abortion, and IPV underlie concerns that restricting access to abortion could increase IPV prevalence around the time of pregnancy, especially among Black pregnant people (14).

Clarifying the relationships between abortion access, IPV or non-intimate DV (IPV/DV), and racial disparities in birth outcomes reached new urgency following the June 2022 U.S. Supreme Court's *Dobbs v. Jackson Women's Health Organization* decision*.* As of November 2023, 18 states enacted complete bans, or gestational age cutoffs at 12 weeks or less—severely restricting abortion access for nearly half of the United States. Although years may pass before the health consequences of complete bans become clear, even before the *Dobbs* decision, many states enacted policies that created barriers to abortion services, such as physician and hospital requirements, gestational limits, funding restrictions, and waiting periods (15). Even these less severe restrictions have been associated with decreases in abortion rates (16) and increases in suicide rates (17), but their relationship to IPV and DV is unknown. Moreover, although often asserted, it is unknown whether states with and without highly restrictive abortion policies exhibit differential racial disparities in IPV. This study aimed to characterize rates of IPV/DV around the time of pregnancy among Black and White individuals living in states with highly restrictive abortion policies compared to their counterparts living in less restrictive states.

## 2 Materials and methods

We analyzed data from 36 states participating in the Centers for Disease Control and Prevention (CDC) Pregnancy Risk Assessment Monitoring System (PRAMS) in 2020. PRAMS is a state-based surveillance system of maternal behaviors, attitudes, and experiences around the time of pregnancy conducted by the CDC in collaboration with state health departments (18). The Institutional Review Board at the University of Michigan approved this study (HUM00204182).

Our cohort represents 1,931,458 deliveries of which 1,368,237 deliveries (70.84%) are from non-Hispanic Black and White birthing individuals. We used two PRAMS core questions to identify individuals experiencing IPV/DV: “In the 12 months before you got pregnant with your new baby, did any of the following people push, hit, slap, kick, choke, or physically hurt you in any other way?” and “During your most recent pregnancy, did any of the following people push, hit, slap, kick, choke, or physically hurt you in any other way?” Participants who (1) selected “yes” to either core question and (2) identified husband or partner, ex-husband or ex-partner, or another family member were classified as experiencing IPV/DV.

We defined pregnancy intention by categorizing participants into two groups based on their responses to the PRAMS core question: “Thinking back to *just before* you got pregnant with your new baby, how did you feel about becoming pregnant? Check ONE answer.” If the participants selected “I wanted to be pregnant later,” “I didn't want to be pregnant then or at any time in the future,” or “I wasn't sure what I wanted,” we categorized them as *not intended* pregnancy. If the participants selected “I wanted to be pregnant then” or “I wanted to be pregnant sooner,” we categorized them as *intended* pregnancy.

**Figure 1:**
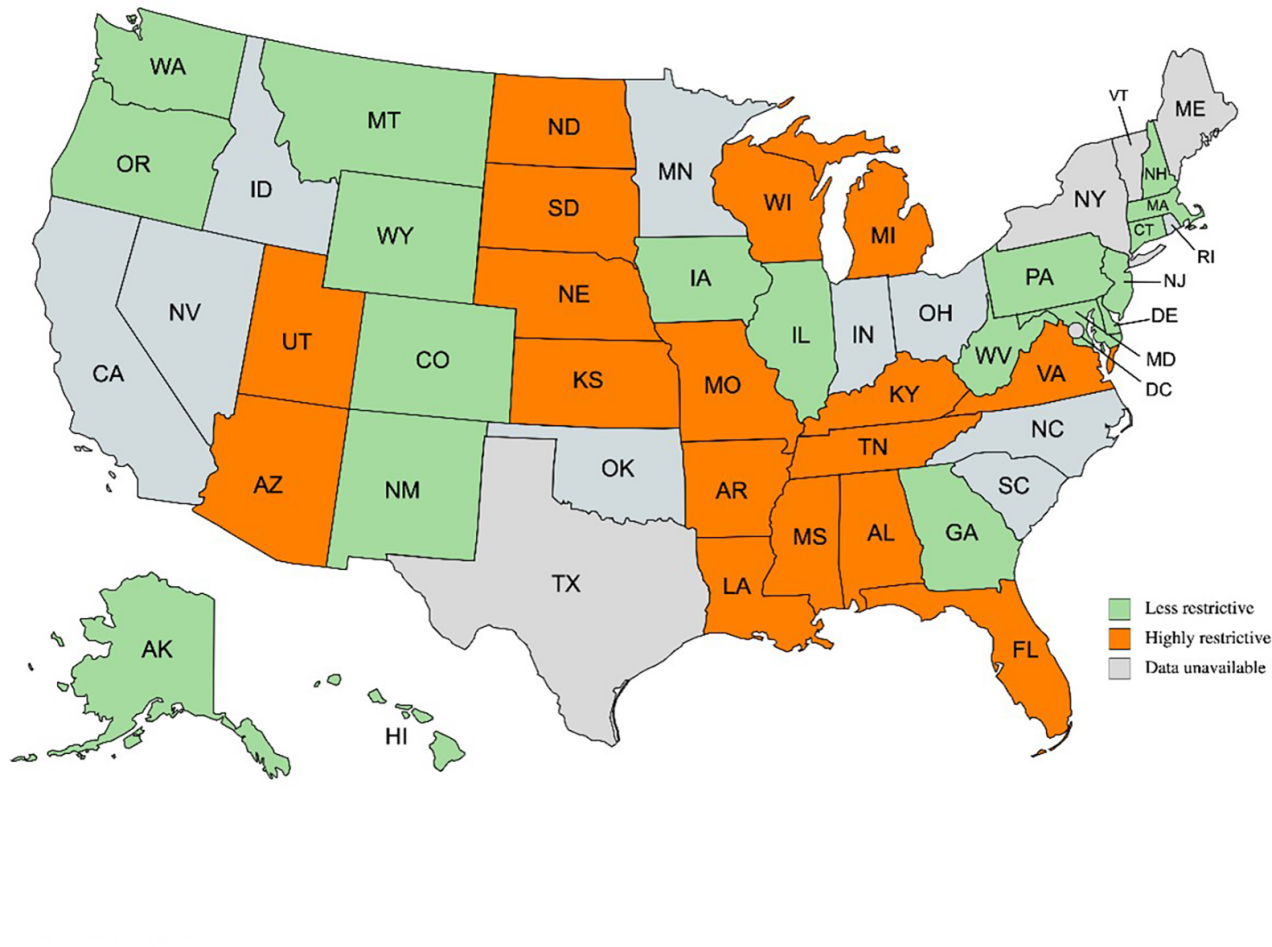
Map of states with PRAMS data available in 2020 categorized into highly restrictive and less restrictive to abortion (19). PRAMS, pregnancy risk assessment monitoring system. Created with mapchart.net.

Archived annual state-level policy data on abortion restrictions were obtained from the Guttmacher Institute. We divided states into highly restrictive (*N* = 17) and less restrictive (*N* = 19) categories using a modified version of the Guttmacher Abortion Policy Hostility Index from 2019 to 2020 (Figure 1). The modified index assigned each state an annual score ranging from 0 to 10 based on the presence of restrictive policies, including gestational limits, inaccurate or misleading counseling requirements, mandatory in-person counseling followed by a waiting period, ultrasound mandates, insurance coverage restrictions, medication abortion restrictions such as telemedicine bans, parental involvement laws, and Targeted Regulation of Abortion Providers (TRAP) laws. A score greater than or equal to 6 indicates that the state is extremely hostile to abortion rights (19).

Initially, we summarized weighted demographic characteristics by state restrictiveness, IPV/DV status, and Black or White race. Next, we used weighted logistic regression to assess the relationship between state abortion restrictiveness and self-reported physical IPV/DV overall and stratified by race. In the overall model, we adjusted for race, marital status, age, education, and insurance coverage. Since income has high rates of missingness in our sample, we used insurance coverage as a proxy for income. We also tested for a statistical interaction between state restrictiveness and race and did not find a significant interaction (*p* > 0.10). To further examine the relationships between state restrictiveness, race, and IPV/DV around the time of a pregnancy, we conducted stratified models by race, adjusting for age, education, insurance coverage, and marital status. To explore the influence of pregnancy intention on the relationship between state restrictiveness and IPV/DV, we ran each model again adjusting for pregnancy intention. We performed all data management using SAS, version 9.4 (SAS Institute) and Stata/SE, version 15.1 (StataCorp) and all statistical analyses in SAS, version 9.4.

## 3 Results

Among the 1,368,237 Black and White birthing individuals in our sample, 50,418 (3.8%) reported experiencing IPV/DV in the period beginning 12 months prior to their most recent delivery through the postpartum period. A higher percentage of both Black and White individuals living in highly restrictive states reported experiencing IPV/DV compared to their counterparts in less restrictive states. Within both highly restrictive and less restrictive states, a higher percentage of Black individuals reported experiencing IPV/DV compared to White individuals (6.6% vs. 4.0%, respectively in highly restrictive states; 3.6% vs. 2.4%, respectively in less restrictive states). The weighted demographic characteristics of our study sample appear in Table 1.

**Table 1:** Weighted demographic frequencies by state restrictiveness, IPV/DV, and race, black and white birthing individuals, United States, 2020.

Characteristic	Highly Restrictive State (*N* = 740,649)				Less Restrictive State (*N* = 627,589)			
	Any IPV/DV^a^ (*N* = 33,969)		No IPV/DV (*N* = 706,680)		Any IPV/DV (*N* = 16,449)		No IPV/DV (*N* = 611,139)	
	Black	White	Black	White	Black	White	Black	White
Weighted Sample^b^	10,854 (6.6)	23,114 (4.0)	154,845 (93.5)	551,835 (96.0)	4,590 (3.6)	11,859 (2.4)	124,095 (96.4)	487,044 (97.6)
Age group, years								
15–19	784 (7.2)	1,719 (7.4)	11,435 (7.4)	18,207 (3.3)	320 (7.0)	1,495 (12.6)	7,616 (6.1)	10,543 (2.2)
20–24	3,092 (28.5)	8,571 (37.1)	39,554 (25.5)	92,946 (16.8)	1,613 (35.1)	2,910 (24.5)	24,748 (19.9)	62,555 (12.8)
25–29	3,095 (28.5)	7,404 (32.0)	45,157 (29.2)	165,116 (29.9)	1,180 (25.7)	2,709 (22.8)	38,396 (30.9)	129,592 (26.6)
30–34	2,349 (21.6)	3,534 (15.3)	36,730 (23.7)	179,108 (32.5)	997 (21.7)	3,365 (28.4)	29,068 (23.4)	174,038 (35.7)
35+	1,534 (14.1)	1,887 (8.2)	21,963 (14.2)	96,451 (17.5)	479 (10.4)	1,380 (11.6)	24,268 (19.6)	110,316 (22.7)
Education								
<High School	1,136 (10.5)	3,978 (17.2)	17,878 (11.6)	35,191 (6.4)	582 (12.7)	2,110 (17.8)	11,232 (9.1)	26,804 (5.5)
High School	5,187 (47.8)	8,715 (37.7)	63,516 (41.0)	127,913 (23.2)	1,684 (36.7)	3,732 (31.5)	41,589 (33.5)	90,015 (18.5)
>High school	4,478 (41.3)	10,420 (45.1)	72,304 (46.7)	387,112 (70.2)	2,305 (50.2)	5,846 (49.3)	70,278 (56.6)	367,694 (75.5)
Marital status								
Married	1,254 (11.6)	5,789 (25.0)	41,299 (26.7)	393,973 (71.4)	881 (19.2)	4,019 (33.9)	42,351 (34.1)	360,881 (74.1)
Not married	9,600 (88.5)	17,326 (75.0)	113,527 (73.3)	157,605 (28.6)	3,709 (80.8)	7,840 (66.1)	81,684 (65.8)	125,948 (25.9)
Insurance								
Medicaid	8,979 (82.7)	15,668 (67.8)	107,609 (69.5)	167,747 (30.4)	3,335 (72.7)	6,739 (56.8)	78,284 (63.1)	124,062 (25.5)
Private	1,690 (15.6)	6,971 (30.2)	44,197 (28.5)	365,643 (66.3)	1,162 (25.3)	4,978 (42.0)	43,382 (35.0)	345,525 (70.9)
No Insurance	186 (1.7)	311 (1.4)	2,780 (1.8)	15,635 (2.8)	84 (1.8)	143 (1.2)	2,096 (1.7)	13,277 (2.7)
Poverty status								
≤138% FPL^c^	7,366 (67.9)	15,214 (65.8)	77,510 (50.1)	129,403 (23.5)	2,381 (51.9)	6,127 (51.7)	52,950 (42.7)	84,059 (17.3)
139–199% FPL	805 (7.4)	2,763 (12.0)	17,537 (11.3)	56,768 (10.3)	345 (7.5)	789 (6.7)	13,347 (10.8)	37,063 (7.6)
200–399% FPL	1,270 (11.7)	2,183 (9.4)	25,514 (16.5)	182,758 (33.1)	523 (11.4)	2,340 (19.7)	25,651 (20.7)	142,508 (29.3)
≥400% FPL	159 (1.5)	1,075 (4.7)	8,829 (5.7)	150,602 (27.3)	386 (8.4)	1,989 (16.8)	12,081 (9.7)	190,696 (39.2)
Pregnancy intention								
Intended	3,056 (28.2)	7,594 (32.9)	54,005 (34.9)	364,250 (66.0)	839 (18.3)	4,559 (38.4)	62,848 (50.6)	346,609 (71.2)
Not Intended	7,799 (71.9)	15,520 (67.2)	100,840 (65.1)	187,585 (34.0)	3,752 (81.7)	7,300 (61.6)	61,248 (49.4)	140,435 (28.8)

^a^ IPV/DV, intimate partner violence or non-intimate domestic violence.

^b^ Data presented as *n* (%); the percentages within this row can be interpreted as the following: 6.6% of Black individuals in highly restrictive states experience IPV/DV. Therefore, the two Black individual columns under highly restrictive states will sum to 100%.

^c^ FPL, federal poverty level.

Overall, we found that birthing individuals are more likely to experience IPV/DV when living in highly restrictive states compared to less restrictive states (aOR: 1.36, 95% CI: 1.15–1.60) (Table 2). For context, this corresponds to a predicted probability for IPV/DV of 3.27% in less restrictive states and 4.93% in highly restrictive states.

**Table 2:** Weighted IPV/DV logistic regression models among all races, with and without pregnancy intention, United States, 2020.

Covariate	Unadjusted Odds Ratio (95% CI)^a^	Adjusted Odds Ratio (95% CI)^a^	Adjusted Odds Ratio (95% CI)^a^
State Restrictiveness			
Highly Restrictive vs. Less Restrictive	**1.50 (1.27–1.76)**	**1.36** **(1.15–1.60)**	**1.31** **(1.11–1.55)**
Race^b^			
American Indian/Alaskan Native vs. Non-Hispanic White		**2.21** **(1.18–4.13)**	**2.21** **(1.20–4.03)**
Asian Pacific Islander vs. Non-Hispanic White		0.55 (0.28–1.06)	0.53 (0.27–1.03)
Black Non-Hispanic vs. Non-Hispanic White		**1.73** **(1.09–2.73)**	1.52 (0.96–2.41)
Hispanic vs. Non-Hispanic White		**1.83** **(1.18–2.84)**	1.70 (0.91–2.62)
Other or Mixed Race vs. Non-Hispanic White		**1.91** **(1.01–3.61)**	1.80 (0.94–3.44)
Missing/Unknown vs. Non-Hispanic White		1.75 (0.79–3.88)	1.74 (0.78–3.88)
Marital Status^b^			
Not Married vs. Married		**4.65** **(3.45–6.26)**	**3.88** **(2.88–5.23)**
Race × Marital Status^b^			
American Indian/Alaskan Native × Marital Status		0.65 (0.31–1.38)	0.65 (0.31–1.34)
Asian Pacific Islander × Marital Status		1.11 (0.43–2.87)	1.08 (0.42–2.81)
Black Non-Hispanic × Marital Status		**0.42** **(0.25–0.69)**	**0.45** **(0.27–0.75)**
Hispanic × Marital Status		**0.40** **(0.24–0.67)**	**0.45** **(0.27–0.74)**
Other or Mixed Race × Marital Status		0.67 (0.32–1.43)	0.69 (0.33–1.48)
Missing/Unknown × Marital Status		0.37 (0.09–1.41)	0.36 (0.09–1.38)
Pregnancy			
Not Intended vs. Intended		–	**2.02** **(1.66–2.45)**
Age			
Age 20–24 vs. ≤19		1.31 (0.95–1.80)	**1.39** **(1.01–1.91)**
Age 25–29 vs. ≤19		1.01 (0.72–1.41)	1.10 (0.78–1.55)
Age 30–34 vs. ≤19		0.91 (0.63–1.30)	0.98 (0.69–1.41)
Age 35+ vs. ≤19		0.83 (0.56–1.21)	0.90 (0.61–1.32)
Education			
Less than High School vs. High School		1.10 (0.82–1.37)	1.03 (0.79–1.34)
More than High School vs. High School		0.98 (0.79–1.21)	1.00 (0.82–1.24)
Insurance			
Medicaid vs. Private		**1.66** **(1.32–2.10)**	**1.58** **(1.26–1.98)**
No Insurance vs. Private		0.93 (0.54–1.61)	0.83 (0.48–1.42)

^a^ Bolded 95% CIs indicates statistical significance.

^b^ These covariates and their corresponding interaction term are not directly interpretable. During our exploratory analysis, we found an interaction between race and marital status; therefore, our overall model includes an interaction term.

In our stratified analyses by race, Black individuals living in highly restrictive states had higher odds of experiencing IPV/DV compared to those living in less restrictive states (aOR: 1.75, 95% CI: 1.24–2.47) after controlling for marital status, age, education, and insurance coverage (Table 3). Similarly, White individuals living in highly restrictive states also had elevated odds of experiencing IPV/DV compared to their counterparts living in less restrictive states (aOR: 1.50, 95% CI: 1.17–1.94) (Table 3).

**Table 3:** Weighted IPV/DV logistic regression models stratified by race among black and white birthing individuals, with and without pregnancy, United States, 2020.

Covariate	Black individuals		White individuals	
	Adjusted OR (95% CI)^a^	Adjusted OR (95% CI)^a^	Adjusted OR (95% CI)^a^	Adjusted OR (95% CI)^a^
State restrictiveness				
Highly Restrictive vs. Less Restrictive^b^	**1.75** **(1.24–2.47)**	**1.64** **(1.15–2.34)**	**1.50** **(1.17–1.94)**	**1.48** **(1.15–1.91)**
Marital status				
Not Married vs. Married	**2.24** **(1.35–3.72)**	**2.05** **(1.24–3.39)**	**3.97** **(2.84–5.55)**	**3.28** **(2.35–4.58)**
Pregnancy				
Not Intended vs. Intended	*–*	**1.72** **(1.21–2.43)**	*–*	**2.22** **(1.67–2.95)**
Age				
20–24 vs. ≤19	1.31 (0.74–2.35)	1.32 (0.74–2.38)	1.14 (0.70–1.86)	1.23 (0.75–2.01)
25–29 vs. ≤19	1.07 (0.60–1.89)	1.12 (0.63–1.99)	0.93 (0.54–1.59)	1.05 (0.61–1.82)
30–34 vs. ≤19	1.24 (0.67–2.28)	1.31 (0.71–2.42)	0.75 (0.43–1.31)	0.83 (0.47–1.44)
35+ vs. ≤19	1.24 (0.61–2.52)	1.31 (0.64–2.68)	0.56 (0.30–1.04)	0.62 (0.33–1.15)
Education				
<High School vs. High School	0.88 (0.54–1.43)	0.88 (0.54–1.43)	1.46 (0.99–2.17)	1.46 (0.98–2.17)
>High School vs High School	1.00 (0.71–1.41)	1.00 (0.71–1.42)	0.93 (0.67–1.29)	0.97 (0.70–1.34)
Insurance				
Medicaid vs. Private	**1.60** **(1.06–2.41)**	**1.57** **(1.04–2.37)**	**1.84** **(1.30–2.60)**	**1.72** **(1.23–2.42)**
No Insurance vs. Private	1.44 (0.40–5.11)	1.45 (0.42–5.07)	0.77 (0.26–2.23)	0.73 (0.25–2.12)

^a^ Bold 95% CIs indicate statistical significance.

^b^ Unadjusted ORs (95% CI): Black individuals, **1.90 (1.35–2.65)**; White individuals, **1.72 (1.34–2.20)**.

Pregnancy intention status was significantly associated with experiencing IPV/DV in the overall and stratified models (Tables 2, 3). The inclusion of pregnancy intention status in our models resulted in a small decrease in the overall odds ratio characterizing the relationship between state restrictiveness and IPV/DV (aOR: 1.36; 95% CI: 1.15–1.60] vs. aOR: 1.31; 95% CI: 1.11–1.55) (Table 2). In our stratified models, the inclusion of pregnancy intention status resulted in a larger magnitude of change among Black delivering individuals, where the adjusted odds ratio of experiencing IPV/DV decreased from 1.75 (95% CI: 1.24–2.47) to 1.64 (95% CI: 1.15–2.34) (Table 3). The adjusted odds ratio among White individuals also decreased, to a smaller degree, from 1.50 (95% CI: 1.17–1.94) to 1.48 (95% CI: 1.15–1.91) (Table 3).

## 4 Discussion

Even prior to the enactment of total or near total abortion bans, individuals living in states with higher levels of abortion restrictions had 50%–75% higher odds of experiencing IPV/DV around their most recent birth. Further, the magnitude of this relationship was greater for Black individuals than White individuals (aORs = 1.75 and 1.50, respectively). These findings provide further evidence for concerns that abortion bans are associated with greater health disparities and higher rates of IPV for Black individuals.

Although we cannot determine whether the observed association is causal in nature, we hypothesize multiple potential pathways could potentially explain the amplified chance of experiencing IPV during pregnancy and living in a restrictive state. First, individuals who would like to obtain an abortion because they are in a violent relationship may not be able to access one because of restrictions. Even prior to the *Dobbs* decision, restrictive abortion policies were associated with decreased rates of abortion and a higher likelihood of pregnancy continuation (16, 20). Prior studies consistently demonstrate that people undergoing abortion report higher rates of IPV (11, 12, 21), and that partner conflict is often a factor in the decision to seek an abortion (13). The Turnaway Study, a landmark study of the consequences of denied abortion, found that individuals unable to obtain a wanted abortion were slower to end a violent relationship and more likely to continue to experience violence compared to those who obtained an abortion (22). Consistent with previous work, we found evidence that pregnancy intention underlies some of the relationship between state abortion policy and IPV, especially among Black delivering individuals.

Second, the observed association between abortion restrictiveness and IPV/DV may also be due to fewer policies or programs aimed at supporting the health and well-being of pregnant people and their families in states that restrict abortions. For instance, restrictive states tend not to have expanded Medicaid, which may result in decreased access to prenatal care and family planning services (23). As noted in an amicus brief filed by hundreds of public health organizations for Jackson Women's Health Organization, the 14 states with the most severely restrictive abortion policies also have the worst health outcomes for birthing people and infants (24).

Third, states that restrict abortion may have fewer policies or state laws that protect people experiencing IPV. For example, although most U.S. states have enacted some form of firearm restriction laws for IPV perpetrators, over half of the states that banned abortion have no domestic violence-related gun regulations. Nonfatal gun use in IPV is common (25) and is used to facilitate coercive control (26), including as a means to ensure the relationship continues.

We focused on disparities in IPV/DV between Black and White individuals in this study. Black individuals are more likely than White individuals to experience reproductive coercion, IPV, and unintended pregnancy (27, 28). Socioeconomic factors such as poverty also disproportionately impact Black individuals, increasing their vulnerability to violent relationships (29). Among Black individuals, IPV is associated with negative physical health outcomes, mental health conditions, and sexual and reproductive health outcomes (30). However, we recognize that other groups experience high rates of IPV and its consequences as well, especially teens, Native American/Alaskan, non-binary, and LGBTQ + individuals (1). These populations are more likely to experience IPV/DV and may experience additional structural and cultural barriers to both health care and legal support. Future studies that are sufficiently powered to examine the relationship between abortion restrictions and IPV among other groups of at-risk people are urgently needed.

This study contributes to a growing body of literature that asserts access to abortion may represent an important structural determinant of health, particularly given the known potential negative health consequences of exposure to IPV/DV during pregnancy (23). It highlights that restrictive abortion policies and higher rates of IPV are clustered in the same states, as well as that the magnitude of the relationship between state restrictiveness and IPV/DV was larger for Black individuals than for White individuals. This is consistent with related literature that identifies racism as a fundamental cause of adverse health outcomes, which acts through multiple, overlapping pathways including structural barriers, cultural racism, and discrimination (31). Within the context of this analysis, racism reduces access to health care including reproductive health services, legal protections that may support individuals experiences IPV/DV, and resources which may help obtain both reproductive health services and protection against IPV/DV. As racial disparities in maternal mortality worsen in the United States, further understanding of these complex dynamics is essential for advancing health equity.

Restrictive abortion policies, IPV/DV, and structural racism directly impact patient care and outcomes. Therefore, health care providers and health care systems must actively recognize and navigate these barriers to improve health outcomes. For instance, in 2012, ACOG recommended routine IPV/DV screening for all perinatal patients (33). Additionally, the Alliance for Innovation on Maternal Health Community Care Initiative (AIM CCI) offers an IPV/DV safety bundle which includes recommendations for culturally appropriate screening and intervention to assuage racial disparities in IPV/DV (34). Provider awareness that individuals experiencing restricted abortion access, as well as structural racism, could be at increased risk of experiencing IPV/DV and its consequences is crucial to protect the health of women.

The key strengths of this study include that PRAMS allows for birthing population estimates at the state level due to its sampling design, inclusion of three-quarters of states, and inclusion of core questions that address IPV/DV victimization. There are, however, several limitations. First, we cannot establish causal relationships between state abortion restrictiveness and IPV/DV using a cross-sectional design. It is likely that there are unaccounted for state-level factors that confound the relationship between abortion restrictiveness and IPV/DV. Specifically, state-level policies regarding mandatory reporting, no-fault divorce, and protective orders may further influence these factors. It is also likely that our study generated conservative estimates of IPV/DV because PRAMS utilizes self-reporting, which may result in underreporting. IPV/DV represents a highly sensitive topic, and respondents may not fully report IPV/DV experiences due to stigma or fear, in addition to common survey limitations such as recall bias. Furthermore, PRAMS does not collect information on psychological violence. Our analysis explores racial trends among Black and White individuals, however we were unable to account for additional or intersecting identities such as gender identity, sexual orientation, and immigration status, which may further impact both risk of IPV/DV and impact of abortion restrictions. Similarly, there is likely within-group heterogeneity among the racial categories used in this analysis, particularly regarding Black subgroups such as African Americans, African immigrants, and Caribbean immigrants. While our data do not support these subgroup analyses, future research should further explore how IPV/DV and abortion restrictions differentially impact other marginalized communities.

Our study included data collected prior to the most extreme category of abortion restrictions— bans or near bans, which may have stronger associations with IPV/DV during pregnancy. Future studies should explore the impact of bans or near bans on the prevalence of IPV/DV and its consequences, including maternal morbidity and mortality. Further, studies should specifically monitor the impact among groups who experience IPV/DV and its health consequences at already high rates, including Black individuals.

## 5 Conclusion

Our finding that even prior to the enactment of abortion bans, individuals in states with more restricted access to abortion were more likely to report IPV/DV alerts us to another possible detrimental impact of abortion restrictions on health. Furthermore, the observation that the magnitude of this relationship was greater among Black individuals than White individuals raises concern that abortion restrictions have the potential to worsen existing health inequities. To translate these findings into action, policymakers should support efforts to address IPV, including wide-ranging approaches such as improving access to education, reducing poverty, implementing and enforcing restraining orders, and offering no-fault divorce (32) — particularly in highly restrictive states. Since Black individuals face greater risk, policies should also comprehensively address the specific needs of this population, including addressing structural barriers to seeking care such as historical racism, perceived discrimination, and medical distrust (30). Health care providers play a critical role in identifying and intervening on IPV/DV, while health care systems may help reduce structural barriers to care and advocate for appropriate health care policy. Finally, future research is needed to determine if abortion restrictions disproportionately impact IPV/DV rates among other vulnerable subpopulations. The relationship between abortion restrictions, IPV/DV, and race remains complex and demonstrates the importance of ensuring access to reproductive health care in all populations.

## Data Availability

The data analyzed in this study is subject to the following licenses/restrictions: The datasets analyzed for this study are available to researchers via request to the Center for Disease Control and Prevention's (CDC's) Pregnancy Risk Assessment Monitoring System (PRAMS). Requests to access these datasets should be directed to https://www.cdc.gov/prams/php/data-research/index.html.
